# Deep Reinforcement Learning for Data Association in Cell Tracking

**DOI:** 10.3389/fbioe.2020.00298

**Published:** 2020-04-09

**Authors:** Junjie Wang, Xiaohong Su, Lingling Zhao, Jun Zhang

**Affiliations:** ^1^School of Computer Science and Technology, Harbin Institute of Technology, Harbin, China; ^2^Department of Rehabilitation, Heilongjiang Province Land Reclamation Headquarters General Hospital, Harbin, China

**Keywords:** cell tracking, linear assignment problem, deep learning, deep reinforcement learning, data association, residual CNN

## Abstract

Accurate target detection and association are vital for the development of reliable target tracking, especially for cell tracking based on microscopy images due to the similarity of cells. We propose a deep reinforcement learning method to associate the detected targets between frames. According to the dynamic model of each target, the cost matrix is produced by conjointly considering various features of targets and then used as the input of a neural network. The proposed neural network is trained using reinforcement learning to predict a distribution over the association solution. Furthermore, we design a residual convolutional neural network that results in more efficient learning. We validate our method on two applications: the multiple target tracking simulation and the ISBI cell tracking. The results demonstrate that our approach based on reinforcement learning techniques could effectively track targets following different motion patterns and show competitive results.

## 1. Introduction

Tracking individual cells in a group is the fundamental of many biomedical analysis tasks, including understanding how genotypes are related to phenotypes, tracking the early development of organs and meristems, and potentially tracking the development of cancerous tumors (Cheng et al., 2019, 2020; Han et al., 2019; Hu et al., 2019). It is often necessary to identify individual cells and follow them over time to gain biological insights from time-lapse microscopy recordings of cell behavior. Microscopic target tracking can provide technical support for the analysis of other features in biological and medical research (Cheng, 2019; Zhao et al., 2020). Therefore, it is of great significance to find an automatic and reliable way to track multiple cells.

There are many procedures and methods for tracking objects at the microscopic level. Tracking-by-detection methods are widely used in multi-target tracking, in which detection and association are two primary issues. Extensive research efforts have focused on detection, especially in cell-tracking applications. For target association between frames, the naïve nearest-neighbor method is commonly adopted but provides unsatisfactory association accuracy. Target association is a combinatorial optimization problem, which is widely studied in computer science and mathematics and many such problems are NP-hard. In general, the linear assignment problem is to find the optimal assignment that maximizes or minimizes the sum of the costs in a cost matrix. Classic algorithms for the linear assignment include the Hungarian method (Kuhn, 1955), auction algorithms (Bertsekas, 1992), and certain variant algorithms.

Recently, some data-driven methods have been proposed to solve combinatorial problems. Vinyals et al. (2015) first proposed a pointer network (PN) to solve combinatorial problems such as the traveling salesman problem and convex hulls. Inspired by the PN, in Milan et al. (2017), a recurrent neural network is used to find the marginal probability based on the cost matrix. A deep Hungarian network based on the recurrent neural network has also been proposed for multi-object tracking (Xu et al., 2019).

Substantial progress in artificial intelligence has been made in supervised learning, where systems are trained on vast amounts of labeled data (Peng et al., 2019a,b,c, 2020). However, supervised learning predominantly works in domains with an abundance of human-labeled data. In many challenging domains, supervised learning fails due to a lack of available data. Reinforcement learning (RL) seeks to create intelligent agents that adapt to an environment by analyzing their own experiences. Bello et al. (2016) and Khalil et al. (2017) suggested using the RL method to train the network without the ground-truth labels. Because it is difficult to obtain optimal solutions for certain NP-hard combinatorial problems. RL is a branch of machine learning that focuses on obtaining an optimal policy to solve specific problems. Following the work of Bello et al. (2016), some researchers have proposed different deep reinforcement learning (DRL)-based methods for solving combinatorial problems that have yielded good performance (Emami and Ranka, 2018; Nazari et al., 2018; Fu et al., 2019).

This work is motivated by several recent proposed DRL methods for NP-hard problems. We propose a DRL approach to automatically search for assignment solutions for a given cost matrix. Specifically, we first modeled the association of cells between frame as an linear assignment problem and formulated the assignment problem with the one-to-one constraint as a DRL problem. Then, with the objective of minimizing the sum of the assignment costs, we used DRL to obtain the optimal assignment solution. To convert the cost matrix into a finite action space, we employ the residual learning and convolutional neural network (CNN) to extract features from a set of training samples and use the pointing mechanism (Bello et al., 2016) to satisfy the one-to-one constraints of the linear assignment problem. Then, the CNN is trained with the REINFORCE algorithm (Williams, 1992) to search for assignment solutions, and the sum of the cost matrix of the selected solution is used as a reward to adjust the parameters of the neural networks.

Our contributions are the following:(1) A simple framework for cell detection and association based on the idea of (2) We introduce a formulation that translates the decision making in the linear assignment problem algorithm into an RL problem. (3) We propose a novel neural network architecture that end-to-end maps the inputs to the decision outputs.

The organization of this paper is as follows. Related work is introduced in section 2. The framework of the proposed method and training details are presented in section 3. In section 4, some experiments are conducted to evaluate the performance of our proposed method. The conclusion is given in section 5.

## 2. Related Work

### 2.1. Cell Tracking

A large variety of cell tracking methods have been described in the existing literature. These cell tracking methods can be broadly grouped into two categories: (i) tracking by model evolution and (ii) tracking by detection.

In tracking by model evolution methods, cell segmentation and tracking are solved simultaneously in each frame of a cell video. Typically, these methods are driven by data in some feature space and make a regularity assumption on the smoothness of the curve. In this framework, cells are represented by parametric or implicit active contour models. Parametric models utilize the explicit representations of cell boundaries such as Gaussian Mixture Models (GMM) (Amat et al., 2014), active meshes (Dufour et al., 2010), or active contours (Zimmer et al., 2002). Implicit models often use the level set to represent the cell contours (Dzyubachyk et al., 2010). These cell tracking methods have some shortcomings. For example, the parametric method depends on the chosen parameterization, and the implicit method is computationally expensive.

Existing cell tracking methods generally adopt the tracking by detection strategy. The tracking by detection method typically consists of two stages: the cell detection stage and cell association stage. In the first stage, the cells are detected by image segmentation methods. Subsequently, in the second stage, detected cells are associated with neighboring frames in real-time or all frames offline. Cell detection can be achieved by classic image segmentation algorithms based on intensity features, gradient features, or texture features (Chenouard et al., 2013; Xing and Yang, 2016). Recently, several deep learning approaches have shown significant success in cell segmentation tasks (Ronneberger et al., 2015; Falk et al., 2019; Gupta et al., 2019).

## 3. Methods

In this section we present a tracking by detection approach to construct the cell trajectories from a time-series microscopy image sequence. The framework consists of two modules: cell detection and cell association. The U-Net segmentation method is employed to detect all the cells in each frame, and then we adopt the traditional single hypothesis tracking method with Kalman filter and frame-by-frame data association to produce the cell trajectories.

### 3.1. Initial Cell Segmentation

Ronneberger et al. (2015) proposed a new neural network for cell segmentation, namely U-Net, which has achieved state-of-the-art results on a wide array of biomedical image segmentation tasks (Ronneberger et al., 2015; Falk et al., 2019). Since then, most attempts to improve the performance of cell tracking methods have been based on the U-Net architecture (Li et al., 2018). In our approach, cell segmentation is performed using the U-Net implementation of Ronneberger et al. (2015).

### 3.2. Cell Time-Series Model

In this work, we assume that each cell can be modeled as a discrete-time Markov process:

(1) $$\begin{array}{l} x_{t}=Ax_{t-1}+Q_{t} \end{array}$$

where *A* is the transition matrix and *Q*_*t*_ is the process noise matrix, which follows a Gaussian distribution. Once the detected cells are retrieved, the detection results *Z*_*t*_ can be viewed as the measurements, where each measurement $z_{t}^{i}\in Z_{t}$ is defined as

(2) $$\begin{array}{l} z_{t}^{i}=Hx_{t}^{i}+R_{t} \end{array}$$

A Kalman filter can be adopted to use those cell detection results to predict the state of cells, which can then be used to formulate the cell association between frames as a linear assignment problem.

### 3.3. Deep Reinforcement Learning Based Cell Association

To solve the target association problem by DRL, we present our solution architecture in three parts: (1) Problem Formulation. We formulate the procedure for selecting an assignment solution as an RL problem to associate target states and measurements. (2) Neural network architecture. An end-to-end architecture that maps from the state space to the action space is designed. (3) Training algorithm. We present the RL algorithm used for the policy search.

#### 3.3.1. Problem Formulation

##### 3.3.1.1. The formulation of linear assignment problem

Assume that the cell trajectories can be denoted as a set $\Omega_{t-1}=\left\{\omega_{t-1}^{1},\omega_{t-1}^{2},\ldots ,\omega_{t-1}^{M_{t-1}}\right\}$ at time *t* − 1. Each element of Ω_*t*−1_ corresponds to a cell trajectory. To find their associated new measurements at time step *t*, each trajectory would be predicted by a Kalman filter and then find the possible association between predicted cell states and new measurements. Let the set $B=\left\{{\hat{x}}_{t|t-1}^{1},{\hat{x}}_{t|t-1}^{2}\cdots ,{\hat{x}}_{t|t-1}^{M_{t-1}})\right\}$ represent the predicted states for all the existing cells at time *t* − 1. Then the association mapping from set B to the measurement set $Z_{t}=\left\{z_{t}^{1},z_{t}^{2},\ldots ,z_{t}^{N_{t}}\right\}$ can be treated as an assignment problem.

**Figure 1:**
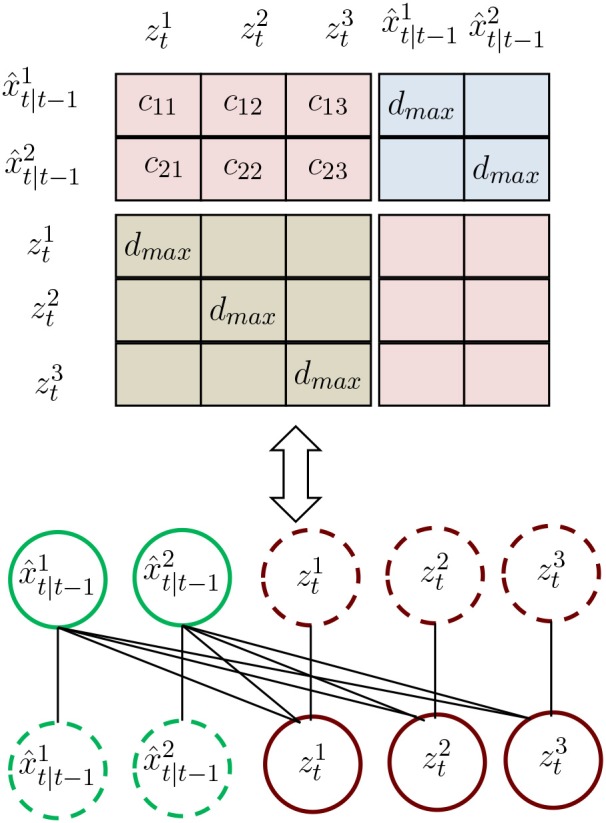
Illustration of the proposed association matrix. ${\left\{{\hat{x}}_{t|t-1}^{i}\right\}}_{i=1}^{2}$ is the prediction by the Kalman filter. ${\left\{{ẑ}_{t}^{i}\right\}}_{i=1}^{3}$ are the measurements.

The values of the cost matrix *D* are calculated through the location distance between the elements of set *B* and the measurements as shown in Figure 1. Unlike the conventional association cost matrix, we construct a new cost matrix that considers the association event. To be specific, matrix *D* is defined as

(3) $$\begin{array}{l} D=\left(\begin{array}{cc} \Lambda & \Upsilon \\ \Gamma & \Lambda^{T} \end{array}\right) \end{array}$$

where *D* is a (*M*_*t*−1_ + *N*_*t*_) × (*M*_*t*−1_ + *N*_*t*_) square matrix, with the row and column indices representing the *M*_*t*−1_ prediction from trajectories and *N*_*t*_ measurements. The matrix *D* consists of four sub-matrices Λ(*M*_*t*−1_ × *N*_*t*_), Υ(*M*_*t*−1_ × *M*_*t*−1_), and Γ(*N*_*t*_ × *N*_*t*_) implies that the corresponding target's state is judged as "Tracked", "Lost," and "New," respectively. In the sub-matrices Υ(*M*_*t*−1_ × *M*_*t*−1_) and Γ(*N*_*t*_ × *N*_*t*_), we define the value of the diagonal element as a distance threshold and other elements to be ∞. Here, when a predicted state is highly self-associated, we consider it to be lost. An estimated state that highly associates itself is considered as a new target. The elements of the sub-matrix Λ(*M*_*t*−1_ × *N*_*t*_) are the distances between the prediction state and measurements.

##### 3.3.1.2. RL formulating for the linear assignment problem

The standard RL formulation starts with an MDP: at time step *t* ≥ 0, an agent is in a state $s_{t}\in S$, takes an action $a_{t}\in A$, receives an instant reward *r*_*t*_ ∈ ℝ and transitions to the next state *s*_*t*+1_ ~ *p*(·|*s*_*t*_, *a*_*t*_). A policy π:S↦P(A) gives a mapping from any state to a distribution over actions π(·|*s*_*t*_). The objective of RL is to search for a policy that maximizes the expected cumulative rewards over a horizon *T*, i.e., $\underset{\pi }{\max}\;J\left(\pi \right):=𝔼\left[\overset{T-1}{\underset{t=0}{\sum }}r_{t}\gamma^{t};\pi \right]$, where γ ∈ (0, 1] is a discount factor and the expectation is w.r.t. randomness in the policy π as well as the environment [e.g., the transition dynamics *p*(·|*s*_*t*_, *a*_*t*_)]. In practice, we consider parameterized policies π_θ_ and aim to find $\theta^{*}=\arg\max J\left(\pi_{\theta }\right)$.

To formulate the procedure of selecting assignment solution algorithms into an MDP, we specify below the state space S, action space A, reward function *r*_*t*_ and transition dynamics *s*_*t*+1_ ~ *p*(·|*s*_*t*_, *a*_*t*_).

**State Space** S. The set of states (S) is defined as all costs of the predicted cell assigned to the detected cell. In this sense, the set S varies according to the number of tasks in the instance.

**Action Space** A. The agent can choose to either assign a predicted cell to a detected cell or not. Thus, we define the action space as A={0,1}, where 1 represents the predicted cell assigned to a detected cell and 0 represents otherwise.

**Reward** *r*_*t*_. For most RL applications, designing a reward function is always a critical part, especially when the agent needs to precisely perform actions in a complicated task. A good reward function will make the agent learn more efficiently and achieve better results. By contrast, an agent with a poor reward function may suffer slow convergence or even produce undesirable results. The objective of the linear assignment problem is to minimize the total cost of the assignment solution. To achieve this objective, we design the reward function as the sum of the assignment cost after producing an assignment solution. Given a cost matrix *C* = {*c*_*ij*_}, *i* = 1, …, *N, j* = 1, …*N* and a selected assignment solution *X* = {*x*_*ij*_}, *i* = 1, …, *N, j* = 1, …*N*, the reward *r*_*t*_ can be defined as $r_{t}=\overset{N}{\underset{i=1}{\sum }}\overset{N}{\underset{j=1}{\sum }}c_{ij}x_{ij}$.

**Transition** *s*_*t*_. In our work, the state transition is deterministic after an action has been chosen because it can directly assign the corresponding task to the person.

#### 3.3.2. Architecture Details

The input of our residual CNN (ResCNN) is a cost matrix *C* that can be treated as the sum of a probability distribution for matching *X* and a noise *V* as

(4) $$\begin{array}{l} C=X+V \end{array}$$

**Figure 2:**
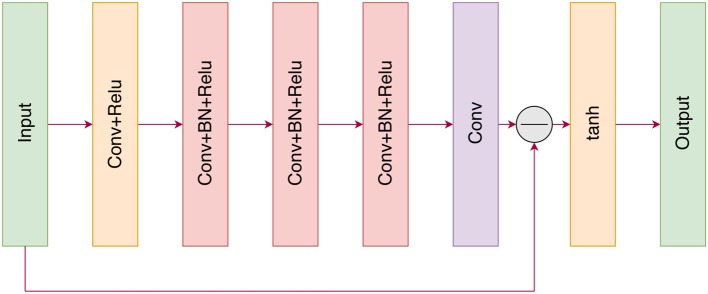
The architecture of the proposed ResCNN network.

The sequence-to-sequence models the linear assignment problem (Milan et al., 2017; Emami and Ranka, 2018) with the aim of learning a mapping function F(C)=X to directly predict the probability distribution. For ResCNN, we adopt the residual learning framework to train a residual mapping R(C)≈V, and then we have X=C-R(C). Figure 2 illustrates the architecture of the proposed ResCNN for learning R(C). In the following, we explain the architecture of ResCNN.

Our proposed neural network is similar to the image denoising network introduced in Zhang et al. (2017). The input of the neural network is a cost matrix that can be regarded as a single-channel image. With the cost matrix *C* as input, the following ResCNN consists of a series of different types of fundamental blocks. The first block consists of a convolution layer (Conv) and a rectified linear unit (ReLU) (Krizhevsky et al., 2012) layer, where the convolution layer utilizes 8 filters of size 3 × 3 × 1 to generate 8 feature maps. Then, the 8 feature maps are fed into three Conv+BN+ReLU-type blocks. For these three blocks, 8 filters of size 3 × 3 × 64 are used, and batch normalization (BN) (Ioffe and Szegedy, 2015) is added between convolution and ReLU. Then, the noise *V* is computed by the last convolution layer, and the probability distribution of the assignment matrix *X* is subtracted from its input (cost matrix). Finally, the probability distribution is clipped by the tanh activation function so that the intensities of the output lie in the range [–1,1].

In summary, the main feature of our ResCNN is the adoption of residual learning to learn R(C) rather than the probability distribution directly. In addition, borrowing the idea of Zhang et al. (2017), batch normalization is incorporated into the ResCNN to speed up the training procedure and improve the performance.

In the following, we will give some important details about our network design and training.

##### 3.3.2.1. Integration of Residual Learning and Batch Normalization

Batch normalization is a standard technique that is widely used in image classification CNN models. Training a deep neural network model is often difficult not only because of the gradient vanishing/exploding problem but also because the distribution of data changes between layers, which is called the “internal covariate shift" phenomenon. Batch normalization is a technique that can relieve this phenomenon by introducing several simple operations to the input data. The goal of the normalization step for batch normalization is to transform the layer input *t* before non-linearity as follows:

(5) $$\begin{array}{l} t^{\prime }=\frac{t-\text{E}\left[t\right]}{\sqrt{\text{Var}\left[t\right]}} \end{array}$$

where E[*t*] and Var[*t*] are the expectation and variance computed over all training data. It is usually impractical to exactly calculate E[*t*] and Var[*t*] with stochastic optimization. Batch normalization instead approximates E[*t*] and Var[*t*] via the mini-batch statistics during training. It would be beneficial if the mini-batch statistics agree well with the full training data statistics.

Batch normalization and residual learning are two important algorithms for designing a neural network architecture. Residual learning and batch normalization can benefit from each other (Zhang et al., 2017). In this paper, we adopt this strategy by integrating these two technologies. Specifically, such an integration not only can significantly increase the training speed but also tends to improve the performance.

##### 3.3.2.2. Zero Padding to Avoid Boundary Artifacts

In the linear assignment problem, the input and output need to be consistent. However, due to the characteristics of convolution, the neural network is prone to producing boundary artifacts without proper handling. There are two common ways to solve this problem: symmetrical padding and zero padding. In our work, we select zero padding to maintain a consistent matrix size.

##### 3.3.2.3. Pointing Mechanism to Satisfy the Constraints

Unlike ordinary visual tasks, for the linear assignment problem, one major characteristic is that one detected cell can only be assigned to one predicted cell. The neural network output should satisfy one-to-one constraints. Let *X* = *C* − *V* denote the outputs of the neural networks. To avoid collisions whereby one task may be assigned to multiple cells simultaneously, we use a mask to set the probability of detected cell that have already been assigned to a predicted cell to −∞, as shown in Equation (6)

(6) $$\begin{array}{l} u_{ij}=\{\begin{array}{ll} Y_{ij} & \text{if}j\neq \pi_{i^{\prime }}\;\forall i^{\prime }<i\\ -\infty & \text{otherwise.} \end{array} \end{array}$$

where *u*_*ij*_ is the probability that predicted cell *i* at time *t* − 1 is assigned to detected cell *j* at time *t*. $\pi_{i^{\prime }}$ is the solution for cell *i*′. Next, a normalized softmax operation is applied to *u* to compute the final output probability matrix.

#### 3.3.3. Training With Policy Gradients

In this paper, we utilize the RL to train the neural network. The input of the network can be denoted as *C* = *c*_*ij*_. The output of the network is the assignment solution π. In this work, we use the sum of the selected costs AC(S|C) as the reward. More specifically, the parameters of the neural network can be denoted as θ, and the goal of training is the expected reward, which is given by an input cost matrix *C* defined as follows:

(7) $$\begin{array}{l} J\left(\theta |C\right)=E_{\pi \sim p\left(\pi |C;\theta \right)}AC\left(\pi |C\right) \end{array}$$

In our work, *p*(π|*C*; θ) is the stochastic policy of a neural network with parameters θ. We learn θ using the Adam optimizer based on the REINFORCE algorithm (Williams, 1992). REINFORCE can make weight adjustments in a direction that lies along the gradient of expected reinforcement. Based on REINFORCE, in each step of training, if the reward, baseline value and probability distribution of prediction are obtained, then the parameters of the neural network, θ, are incremented by an amount

(8) $$\begin{array}{l} \nabla_{\theta }J\left(\theta |C\right)=E_{\pi \sim p_{\theta \left(\cdot |C\right)}}\left[\left(AC\left(\pi |C\right)-b\left(C\right)\right)\nabla_{\theta }\log p_{\theta }\left(\pi |C\right)\right] \end{array}$$

where *b*(*C*) denotes the baseline value of the assignment cost and is used to reduce the variance of the gradients. If we randomly obtain *M* *i.i.d*. samples, then the above gradients can be approximated by

(9) $$\begin{array}{l} \nabla_{\theta }J\left(\theta |C\right)\approx \frac{1}{M}\overset{M}{\underset{i=1}{\sum }}\left[\left(AC\left(\pi_{i}|C_{i}\right)-b\left(C_{i}\right)\right)\nabla_{\theta }\log p_{\theta }\left(\pi_{i}|C_{i}\right)\right] \end{array}$$

For a cost matrix, the baseline value *b*(*C*_*i*_) is initialized by calculating the sum of the cost of the assignment solution that is generated by the neural network. In each step, the baseline value is updated as follows:

(10) $$\begin{array}{l} b^{\prime }\left(C_{i}\right)=b\left(C_{i}\right)+\alpha \left(AC\left(\pi_{i}|C_{i}\right)-b\left(C_{i}\right)\right) \end{array}$$

Algorithm 1 gives the pseudo-code of the training procedure of the neural network.

**Algorithm 1:** Training Procedure

1: Training set ${\left\{C_{i}\right\}}_{i=1}^{M}$, number of training steps *T*, batch size *B*.
2: Initialize the neural net params θ.
3: Initialize baseline value.
4: **for** *t = 1 to T* **do**
5: Select a batch of samples *C*_*i*_ for *i* ∈ {1, ⋯ , *B*}.
6: Sample solution π_*i*_ based on *p*_θ_(·|*C*_*i*_) for *i* ∈ {1, ⋯ , *B*}.
7: Let *g*_θ_= $\frac{1}{B}\overset{B}{\underset{i=1}{\sum }}\left[\left(AC\left(\pi_{i}|C_{i}\right)-b\left(C_{i}\right)\right)\nabla_{\theta }logp_{\theta }\left(\pi_{i}|C_{i}\right)\right]$.
8: Update θ = *ADAM*(θ, *g*_θ_).
9: Update baseline *b*(*C*_*i*_) = *b*(*C*_*i*_)+α(*AC*(π_*i*_|*C*_*i*_) − *b*(*C*_*i*_)) for *i* ∈ {1, ⋯ , *B*}.
10: **end for**
11: return neural net parameters θ.

## 4. Experiments

To evaluate the performance of our proposed method, we consider two applications of the linear assignment problem: maximum weight matching (MWM) and data association for multi-target tracking. We first compare our method with the state-of-the-art DRL method for maximum weight matching. Then, we test our method on a multi-target tracking scenario. Finally, we evaluate our proposed method on three cell microscopy datasets, Fluo-N2DH-GOWT1, PhC-C2DH-U373, and Fluo-N2DH-SIM+ from the ISBI 2015 Cell Tracking Challenge (Maška et al., 2014). Each datasets contains 2 training sequences and 2 challenge sequences. Since it's hard to get the ground truth of segmentation and trajectories in the challenge datasets, we performed tracking experiments on testing datasets.

In all experiments, we used 500, 000 training samples for the data association. To produce the training samples, we randomly sample *M* + *N* points in the euclidean space to simulate the data association between two frames. We use the same hyper-parameters to train our model. The initial learning rate for the Adam optimizer is 10^−3^ and decays every 5,000 steps by a factor of 0.96.

### 4.1. Maximum Weight Matching

Define a weighted bipartite graph *G* = (*V* = {*V*_1_, *V*_2_}, *E*), where *V* is the vertex set containing two disjoint vertex sets *V*_1_ and *V*_2_, with |*V*_1_| = *N* and |*V*_2_| = *N*, and *E* is the set of all edges between every node *v*_1_ ∈ *V*_1_ and *v*_2_ ∈ *V*_2_. Let *w*_*ij*_, *i* ≤ *i* ≤ *N*, 1 ≤ *j* ≤ *N* denote the associated weight for the edges in the graph. Then, a matching in a graph *G* is a subset of *E* such that no two edges share a common vertex. A maximum weight matching is a matching such that the sum of the weights of the edges in the matching is maximal (Emami and Ranka, 2018). In our simulation, each vertex of the graph is represented by a point (*x*_*i*_, *y*_*i*_), and *W*_*ij*_ is the Euclidean distance between vertex *i* and *j*. We select the optimality ratio as $\frac{\text{predicted matching weight}}{\text{optimal matching weight}}\in \left[0,1\right]$ to measure the performance of our proposed method. The optimal matching weight is computed by the Hungarian algorithm, and the predicted matching weight is obtained by our method.

We trained our method on MWM with *N* = {15, 20, 25}. The results are compared with SPG+Matching and AC+Matching (Emami and Ranka, 2018), two DRL method solvers for the MWM problem. The results in Table 1 are the median optimality ratios on the test set. As a baseline, the performances of SPG+Matching and AC+Matching also are presented in Table 1. We observe drastic drops in median optimality ration for the AC+Matching methods with an increasing number of nodes. By contrast, the performances of SPG+Matching and our method show less drastic drops. The results clearly show that our model is competitive with AC+Matching and SPG+Matching methods.

**Table 1:** Median optimality ratios on the MWM test set.

	***N* = 15**	***N* = 20**	***N* = 25**
AC+Matching	0.935	0.897	0.725
SPG+Matching	0.904	0.895	0.889
Ours	0.977	0.968	0.965

### 4.2. Simulated Multiple Target Tracking

**Figure 3:**
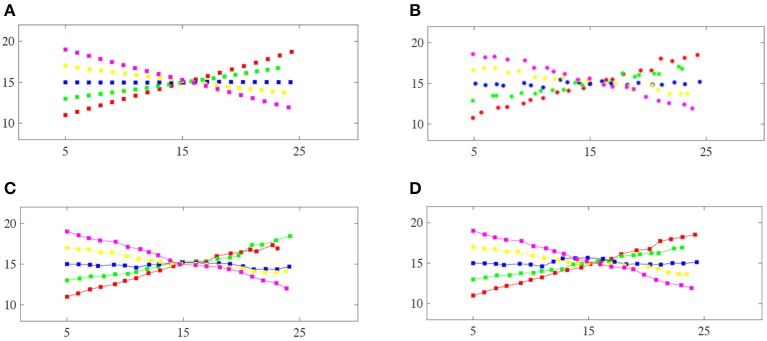
Comparison of the track maintenance performance of different algorithms: **(A)** Ground-truth trajectories of the five targets, **(B)** the measurements of the five targets, **(C)** the JPDA filter, **(D)** our proposed method. Each color corresponds to a particular target. Note that our method correctly resolves this crossing case, whereas the JPDA filter switches the two trajectories after the targets cross.

One major application of linear assignment is data association for multi-target tracking. Therefore, we set up a simulated multi-target tracking scenario to evaluate the performance of the proposed method similar to Milan et al. (2017). Five targets cross each other at a certain time. The track state *x* is represented by a vector $\left[x\;y\;\dot{x}\;\dot{y}\right]$, which contains the position (*x, y*) and velocity (*ẋ*, *ẏ*) information. Figure 3A shows the ground truth of the five targets. The measurements provide noisy positions for the targets, i.e., *z*_*t*_ = *Hx*_*t*_ + *v*_*t*_, where $H=\left[1\;0\right]⊗I_{2\times 2}$ and $v_{t}\sim N\left\{0,R\right\}$. Figure 3B gives the measurements for *R* = 0.05**I**_2 × 2_.

We replace the data association part of JPDA with our method and call it JPDA-RL. The input matrix *C* ∈ ℝ^*N*×*N*^ is the Mahalanobis distance between the estimated target states and the measurements. We compare JPDA-RL with the traditional joint probabilistic data association (JPDA) filter (Fortmann et al., 1980), an approximation of the JPDA filter with the 10 best association hypotheses (Hamid Rezatofighi et al., 2015), an approximation of the JPDA filter with the Hungarian algorithm used to solve the association probabilities and the supervised LSTM used to solve the association problem in Milan et al. (2017).

Figure 3 shows the tracking results from the traditional JPDA filter and our proposed method with the JPDA filter of a single run. The traditional JPDA filter cannot handle the coalescence phenomenon. Our method can correctly distinguish the targets after they have crossed each other.

We employ two metrics to evaluate the tracking results: the Optimal Sub-pattern Assignment metric for track (OSPA-T) and Number of Identity Switch (IDSW). The OSPA-T distance (Ristic et al., 2011) is a metric used to evaluate differences between the real tracks $T_{t}=\left\{X_{t}^{1},\ldots ,X_{t}^{m}\right\}$ and the estimated tracks ${\hat{T}}_{t}=\left\{{\hat{X}}_{t}^{1},\ldots ,{\hat{X}}_{t}^{n}\right\}$ by computing the quantity

(11) $$\begin{array}{l} d_{p}^{\left(c\right)}\left(T_{t},{\hat{T}}_{t}\right)=(\frac{1}{n}(\underset{\pi \in \prod_{n}}{\min}\sum_{i=1}^{m}d^{\left(c\right)}{\left(X_{t}^{i},{\hat{X}}_{t}^{\pi \left(i\right)}\right)}^{p}\\ {\;+c^{p}\left(n-m\right)))}^{1/p}\text{ if }m\leq n\\ d_{p}^{\left(c\right)}\left(T_{t},{\hat{T}}_{t}\right)=d_{p}^{\left(c\right)}\left({\hat{T}}_{t},T_{t}\right)\text{ elsewhere} \end{array}$$

where *d*(·, ·) is the L2-norm, Π_*n*_ is the permutations in {1, …, *n*} and $d^{\left(c\right)}\left(X_{t}^{i},{\hat{X}}_{t}^{j}\right)$ is the distance between $X_{t}^{i}$ and ${\hat{X}}_{t}^{j}$ such that

(12) $$\begin{array}{l} d^{\left(c\right)}\left(X_{t}^{i},{\hat{X}}_{t}^{j}\right)=\min\left(c,d\left(X_{t}^{i},{\hat{X}}_{t}^{j}\right)\right) \end{array}$$

To compute the OSPA-T distance for the estimated tracks and true tracks, two parameters, the cardinality penalty *c* and outlier sensitivity *p*, need to be set. In our simulations, we set *c* = 1 and *p* = 1.

In Table 2, we present a comprehensive comparison of the average OSPA-T distance and IDSW for different algorithms for different measurement noise levels. Interestingly, the IDSW of our method is lower for other algorithms at low measurement noise levels.

**Table 2:** Average OSPA-T distance and IDSW for different methods over 100 random runs.

**Method**	***R* = 0.01I_2_**		**R = 0.05I_2_**		***R* = 0.1I_2_**	
	**OSPA-T**	**IDSW**	**OSPA-T**	**IDSW**	**OSPA-T**	**IDSW**
JPDA	0.19(0.05)	0.90(0.88)	0.34(0.11)	0.70(0.82)	0.41(0.12)	0.40(0.70)
JPDA_10_	0.23(0.10)	0.70(0.67)	0.37(0.11)	0.90(0.88)	0.43(0.09)	1.10(0.99)
JPDA-HA	0.28(0.06)	0.60(0.84)	0.37(0.10)	0.70(0.95)	0.46(0.14)	1.30(0.82)
JPDA-RL	0.28(0.06)	0.60(0.84)	0.36(0.08)	0.60(0.70)	0.45(0.13)	1.10(0.99)
LSTM	0.11(0.01)	1.07(0.84)	0.21(0.01)	1.00(0.74)	0.37(0.11)	0.60(0.89)

*The standard deviations are given in parentheses*.

### 4.3. Cell Tracking

The segmentation task by U-Net and data association by DRL are conducted on AMD Ryzen 9 3900X 12 core processors with a GeForce GTX 2060 graphics card. For comparison, segmentation (SEG), tracking (TRA) accuracy measures and overall performance (OP) are adopted to evaluate the tracking performance. For TRA, Acyclic Oriented Graph Matching (AOGM) is used to count the changes needed to transform the cell tracking family tree into the ground-truth graph. OP is defined as the mean of TRA and SEG.

The results of this work are compared against the best performing available methods for each dataset. For the *Fluo-N2DH-GOWT1-01* dataset, we compare our method with the two tracking-by-detection [*CPN* (Akram et al., 2017) *KTH* (Magnusson and Jaldén, 2012)] and one joint cell detection and tracking [*BLOB* (Akram et al., 2016)] methods as the baselines. For the *PhC-C2DH-U373* dataset, we use the best performing *U-Net* (Ronneberger et al., 2015) and a graph cuts and model evolution-based tracking method (*GC-ME*) (Bensch and Ronneberger, 2015) as the baselines. For the *Fluo-N2DH-SIM+* dataset, we use a Siamese matching-based tracker based on the U-Net segmentation results (*U-Net-S*) (Gupta et al., 2019) as the baseline.

Table 3 lists the TRA, SEG and OPT scores for all methods over three datasets. It can be observed that our method yields the best TRA, SEG and OPT over the *Fluo-N2DH-GOWT1-01* sequence. However, our method has a lower TRA score over the *Fluo-N2DH-GOWT1-02* sequence. One reason for the lower TRA score of our method is that the *Fluo-N2DH-GOWT1-02* sequence has multiple cell events, including mitosis, apoptosis and cell fusion. Our method does not consider the complex process of cell differentiation.

**Table 3:** TRA, SEG and OPT performance for our method, CPN, KTH (Magnusson and Jaldén, 2012), BLOB (Akram et al., 2016), U-Net (Ronneberger et al., 2015), U-Net-S (Gupta et al., 2019), and GC-ME (Bensch and Ronneberger, 2015).

		**TRA**	**SEG**	**OPT**
Fluo-N2DH-GOWT1-01	CPN	0.9864	0.8506	0.9185
	BLOB	0.9733	0.7415	0.8574
	KTH	0.9462	0.6849	0.8155
	Ours	**0.9875**	**0.8585**	**0.9230**
Fluo-N2DH-GOWT1-02	CPN	**0.9719**	0.8725	0.9222
	BLOB	0.9628	0.9046	0.9337
	KTH	0.9452	0.8942	0.9197
	Ours	0.9575	**0.9181**	**0.9378**
PhC-C2DH-U373-01	CPN	0.9594	0.7336	0.8456
	U-Net	0.9869	**0.9375**	**0.9622**
	GC-ME	0.9779	0.8748	0.9264
	Ours	**0.9919**	0.8527	0.9223
PhC-C2DH-U373-02	CPN	0.9346	0.7376	0.8361
	U-Net	**0.9547**	**0.8303**	0.8925
	GC-ME	0.9040	0.7567	0.8304
	Ours	0.9318	0.7735	0.8527
Fluo-N2DH-SIM+-01	U-Net-S	**0.9862**	**0.8866**	**0.9364**
	Ours	0.9841	0.8854	0.9348
Fluo-N2DH-SIM+-02	U-Net-S	0.9597	0.7381	0.8489
	Ours	**0.9618**	**0.7616**	**0.8617**

*The best TRA and SEG values for each sequence are highlighted*.

For the *PhC-C2DH-U373* sequences, the *U-Net* tracking method uses the cell segmentation model trained from two sequences. Therefore, the SEG score of *U-Net* is the best among all algorithms over the *PhC-C2DH-U373* sequences. However, even with that advantage, our method still obtains a higher TRA score on the *PhC-C2DH-U373-01* sequence. *U-Net* produces very accurate cell segmentation masks on *PhC-C2DH-U373* sequences, but for the data association step, it often fails to associate correctly. The reason is that *U-Net* utilizes the greedy search method to link the cell segmentation between frames.

For *Fluo-N2DH-SIM+* sequences, our method has similar performance with *U-Net-S*. Once the cells have been detected, our method for cell tracking is able to achieve high overall accuracy in linking the cells between frames.

## 5. Conclusion

In this paper, we presented a solution to the problem of data association in cell tracking using the deep reinforcement learning. We formulated the data association problem into a linear assignment problem and then proposed a deep reinforcement learning framework which utilizes a residual CNN neural network. In simulation results, we compare the proposed method with other state-of-the-art approaches on various cell tracking datasets, and the results show that the proposed method achieves better comprehensive performance. Thus, our method likely has applications in the field of biomedical engineering. There are also some limitations of our tracking method that leave room for improvement. In future research, we plan to improve the data association method to deal with one-to-many and many-to-one association problems.

## Data Availability Statement

All datasets generated for this study are included in the article/supplementary material.

## Author Contributions

LZ and JW substantially contributed to the conception and design of the study. XS analyzed and interpreted the data. LZ, JW, and JZ drafted the article.

### Conflict of Interest

The authors declare that the research was conducted in the absence of any commercial or financial relationships that could be construed as a potential conflict of interest.
